# The interplay of neural networks enabling social interaction: Protocol of a multi-level, mixed-methods study of empathy and theory of mind

**DOI:** 10.1186/s40359-026-05886-4

**Published:** 2026-11-04

**Authors:** Philipp Kanske, Shuo Chen, Rita Hansl, Andrea Karaiskaki, Nick Michalek, Esther Preuschhof, Katharina Förster, Konrad Lehmann, Lara Z. Maliske

**Affiliations:** 1 Clinical Psychology and Behavioral Neuroscience, Institute of Clinical Psychology and Psychotherapy, Faculty of Psychology, TUD Dresden University of Technology, Dresden, 01187 Germany; 2 Department of Psychology, Faculty of Psychology and Educational Sciences, Babeș- Bolyai University, Cluj-Napoca, Romania; 3 International Max Planck Research School on Cognitive NeuroImaging, Max Planck Institute for Human Cognitive and Brain Sciences, Leipzig, Germany; 4 Child and Adolescent Psychotherapy, Institute of Psychology, Faculty of Psychology and Movement Sciences, Universität Hamburg, Hamburg, Germany

**Keywords:** Empathy, Theory of Mind, Social cognition, Prosociality, fMRI, fNIRS, Hyperscanning, Ecological momentary assessment, Social interaction, Mentalizing

## Abstract

**Background:**

The quality and frequency of social interactions are among the best predictors of long-term mental and physical health. So how do good social interactions arise? Social neuroscience has focused on a number of affective and cognitive functions including empathy and Theory of Mind (ToM) as potential “building blocks” of social interaction. However, utilizing mainly simplistic and passive paradigms, we still fail to describe how actual, live social interactions emerge. Here, the concept of “building blocks” may have become an impediment to progress. Recent evidence suggests that in more complex experimental tasks, socio-affective and -cognitive processes are not simply stacked up, but are dynamically working together, just as the underlying neural networks seem to engage in lively interplay. The main objective of the study presented here (INTERACT) is to understand how adaptive social interactions emerge by elucidating the interplay of networks and processes.

**Methods:**

Using a combination of methods and across data levels, INTERACT will move from the comprehensive investigation of behavioral and neural interaction patterns of social affect and cognition in controlled lab settings to completely free social interactions in people’s everyday lives. First, we adapt an established, behavioral and functional magnetic resonance imaging (fMRI) task measuring empathy and ToM (EmpaToM) to study the **c**onjoint effects of both functions (cEmpaToM) by manipulating their dependencies. Second, we set up an **i**nteractive task version (iEmpaToM) for hyperscanning functional near-infrared spectroscopy (fNIRS) in which participants engage in a conversation with another participant that elicits empathy and ToM. Third, we implement a multi-agent ecological momentary assessment (EMA) setup in which participants and a close social partner complete daily questions about their social interactions triggered by digitally detected physical proximity. Lastly, each of these assessments includes measures of prosocial behavior to investigate how prosociality is impacted by empathy and ToM interactions.

**Discussion:**

Combining a novel experimental approach with single and dual neuroimaging (hyperscanning) and multi-agent EMA, INTERACT will shift from a modular to a complex systems study of actual social interaction. INTERACT aspires to develop an interdisciplinary model of social interactions that expands our knowledge and applies it to real-world settings.

## Background

Imagine living in a society of telepaths, where you readily know everybody’s thoughts and emotions. While this may sound like fantasy or science fiction, humans are actually remarkably good at creating a kind of *representation* of other people’s internal states. We empathically share others’ emotions and take their perspectives – highly adaptive abilities, as knowing about the mental states that drive others’ behavior means you can predict and adjust to it [1–3]. Social neuroscience has extensively investigated these affective and cognitive “building blocks” of sociality and the neural networks that enable them. However, the approach that the field has taken, has treated participants mostly as passive observers of social stimuli [4]. Yet, social interaction is anything but passive. It unfolds dynamically in a mutually dependent and highly complex manner. When we interact, affective and cognitive processes will not function as modular, isolated “building blocks”. Rather, the dynamic flow of events demands affect and cognition to be integrated, tying together different brain networks in a vibrant interplay. Accounting for that interactivity (1) between affect and cognition and (2) between people, may be key to moving forward in our understanding of social behavior. Moreover, social experiences are inherently embodied: interoceptive and other bodily signals shape how individuals perceive, interpret, and respond during social interactions [5, 6]. This is important as – ultimately – human existence is social existence. Encounters with others can be actual life savers; the number of daily social interactions and close friends are two of the best predictors of longevity [7]. Difficulties with social interaction, on the other hand, are involved in most mental disorders [8]. Pandemic times with prolonged physical distancing in the early 2020s have highlighted these relations [9]. To find better ways to improve social capacities, it is now time to build on and eventually replace our static conceptions of social information processing.

T​he overarching goal of the hereby presented INTERACT project is to make the pivotal move to studying the components of social interaction in concert, in complex and dynamically unfolding social situations. The main objectives are (1) to elucidate the interplay of affective and cognitive components of social interaction and the neural networks enabling them, (2) to make critical steps towards doing so in interactive and realistic social encounters and combine these with assessing social interactions in people’s everyday life, (3) to determine how ongoing bodily signals interact with neural processes to inform social engagement and (4) to probe their impact on prosocial behavior. We will elaborate on each of the objectives in detail below.

The first objective of INTERACT is to elucidate the interplay of the neural networks that enable socio-affective and -cognitive processes. Social behavior is likely enabled by a plethora of affective and cognitive functions, including perceptual, attentional, and motivational processes, mirroring, action observation, emotion recognition, person identification, language and many more [10]. How these functions interact in natural social behavior is little understood and potentially highly complex.

**Fig. 1:**
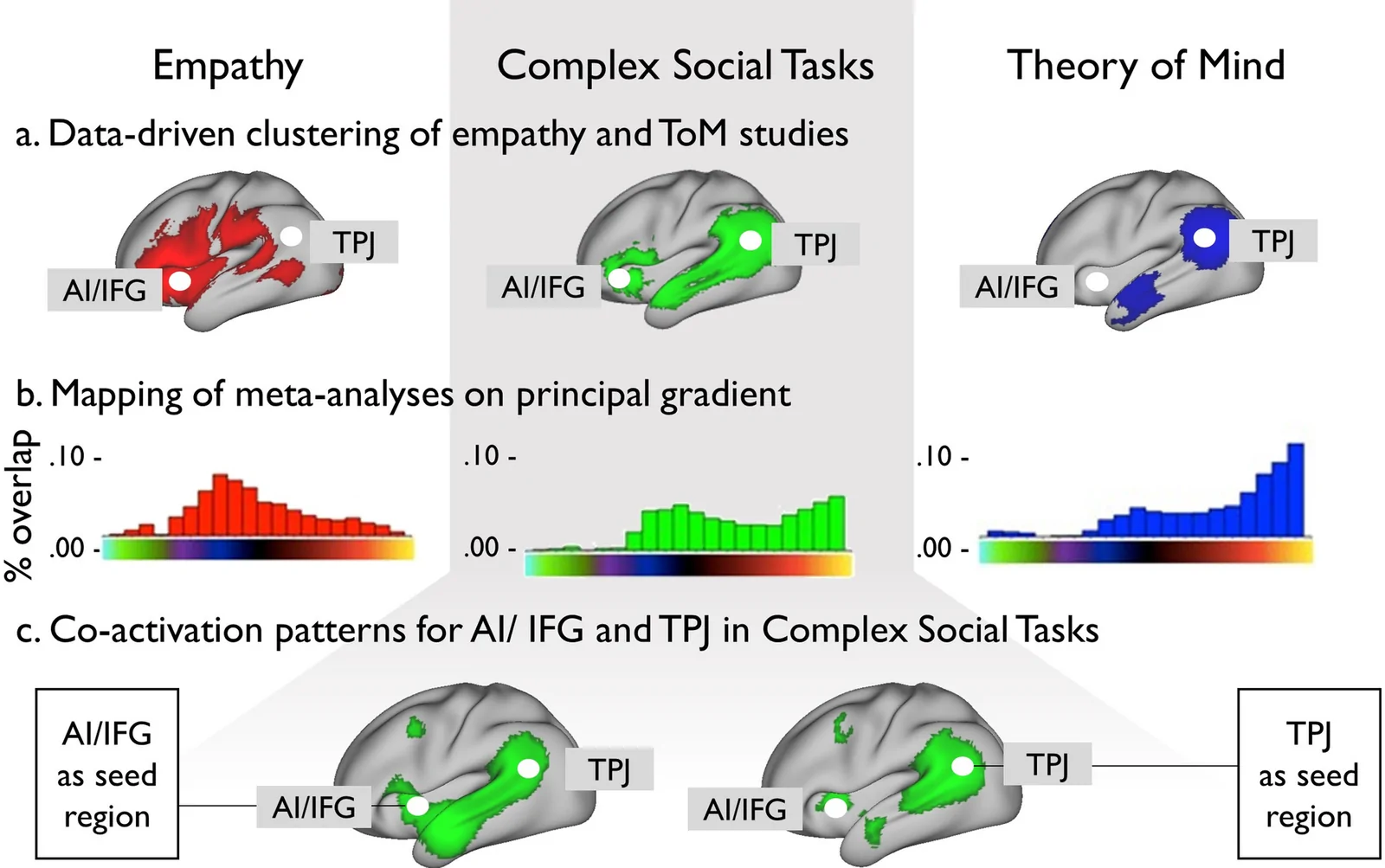
Overview of social affect and cognition related neural networks. (**a**) Meta-analytic clustering yields three clusters associated with classical empathy (control networks, CNs) and ToM studies (default mode network, DMN) as well as a third cluster of more complex social tasks including regions from both empathy and ToM clusters (e.g., AI/IFG and TPJ) [11]. (**b**) Mapping of the clusters on a principal gradient of cortical organization (bar below histograms), ranging from sensory/unimodal (e.g., visual cortex; green) to abstract/transmodal areas (e.g., DMN; yellow), confirms that complex social tasks activate regions of empathy and ToM networks. (**c**) Meta-analytic connectivity modeling with AI/IFG or TPJ as seed regions corroborates the concurrent activation of DMN and CNs for complex social tasks [12]

To move towards interactivity, INTERACT will exemplarily select two particularly high-level functions, the capacity to share others’ emotions (often termed *empathy*, though broader definitions exist) and to take their perspective (also termed *Theory of Mind (ToM)* or *mentalizing*). These functions encompass and result from a number of other social functions, such as emotion recognition or person identification and are of particular significance in that they provide a representation of the mental states of others, the covert driving forces of overt behavior. Their neuroscientific investigation has been extensive and meta-analytic evidence supports the notion that the two concepts can be largely dissociated on a neural level (Fig. 1a, b) [11]. The anterior insula/inferior frontal gyrus (AI/IFG), together with other regions of the salience and other control networks (CNs), are involved in empathy. The temporoparietal junction (TPJ) and other parts of the default mode network (DMN) are primarily recruited during ToM.

​Crucially, the data-driven meta-analytic clustering of​ social tasks also showed that some studies fall into a third category that activates DMN and CNs in parallel. As this finding reveals a previously largely overlooked mode of social brain function, we corroborated that the concurrent activation of DMN and CNs in this category stems from simultaneous activations within the same task in a meta-analytic connectivity modeling analysis (Fig. 1c) [12]. The tasks in this category are more complex and include both empathy and ToM demands. Upon reviewing the few studies that have tested whether and how DMN and CNs communicate during these tasks, the picture is largely inconclusive, but suggests bidirectional excitatory and inhibitory connections [13]. Also, the functional role of the network interactions is unclear. For instance, an inhibitory influence of AI/IFG on TPJ may reflect how excessive emotion sharing hinders understanding of another’s perspective. In other situations, being able to share someone’s emotion may depend on accurately understanding their perspective, consistent with an excitatory influence of TPJ on AI/IFG. The first INTERACT objective is to understand this overlooked mode of integrated social brain functioning, by probing the interplay of empathy- and ToM-related networks and specify their function.

The second objective of INTERACT is to study empathy and ToM in interactive, naturalistic social encounters. The initial steps that neuroscience has taken in the study of social affect and cognition were inevitably reductionist in nature, to identify and isolate its crucial processes [14, 15]. The field then moved to more realistic and ecologically valid tasks, also linking such ecologically valid functional magnetic resonance imaging (fMRI) measures to real world social behavior [16–18]. The next step needs to add the full and actual social interaction, mainly because of two reasons. Discussions around a “2nd-person neuroscience” rightly criticize that in standard tasks, (1) as if behind a one-way mirror, participants perceive a social environment, but are invisible to those in it, precluding any bidirectional information flow. (2) Consequently, participants are deprived of their ability to act with one another, undermining the very idea of inter*action*. These limitations break apart the natural unity between individual and social environment that is key to understanding the mechanisms of social interaction. Despite the well-founded, face-valid criticism, studies aiming to overcome these issues are still the exception to the rule. They do, however, suggest simultaneous DMN and CN involvement in social interaction, although empathy and ToM were not explicitly required [19]. Conceptually, emerging bidirectional information flow between two agents engages ToM capacities not only in taking the other’s perspective, but also for predicting how one’s own behavior is perceived by the other, resulting in nested and recursive cognitive patterns [20]. Similarly, bidirectional information flow also poses demands on empathizing; rather than one agent adopting the emotional state of another, real interaction requires this adoption to be interlocked, mutual, and dynamic. INTERACT’s second objective is to investigate the interplay of empathy and ToM, and their neural correlates, in actual social interactions involving bidirectional information flow and action.

The third objective is to examine how bodily and neural processes interact to guide social behavior. Social interaction is not solely supported by the brain but emerges from continuous interactions between the brain and the body [21, 22]. A growing literature suggests that afferent signals from the cardiovascular, respiratory, and autonomic systems shape emotional experience, attention, decision-making, and social cognition [23]. Interoceptive signals provide the brain with continuous information about the physiological state of the body and are integrated in regions such as the anterior insula and anterior cingulate cortex, which also play central roles in empathy and salience processing [24, 25]. However, most existing studies have investigated brain activity and physiology separately, or in passive observation paradigms, leaving largely unexplored how ongoing bodily signals shape neural processing during dynamic social interactions. Likewise, it remains unclear how moment-to-moment fluctuations in autonomic activity interact with the neural networks supporting empathy and ToM to influence social behavior. By combining neuroimaging methods with psychophysiological recordings, INTERACT will explore the dynamic interactions between bodily signals and neural activity during naturalistic social encounters.

A fourth objective is to link empathy and ToM and their interaction to prosocial behavior - that is, voluntary behavior intended to benefit others. Vast evidence from economics shows that humans are indeed much more prosocial than rational models of behavior suggest [26]. Empathy and ToM seem to be particularly important for acting prosocially (and forbearing antisocial behavior) as understanding another’s reasons and sharing their suffering can be important motivators to help. Also, related to adaptive social functioning more generally, difficulties in social interactions associated with many mental disorders are partly attributable to deficits in empathy and ToM [27]. Using the neural activity patterns associated with empathy and ToM can indeed predict subsequent prosocial acts, although with an accuracy of no more than 10–15% above chance [28]. The issue at hand is that empathy and ToM research on prosocial behavior mainly focused on these functions individually in non-interactive settings. However, particularly in complex situations empathy and ToM may only function in tandem. For instance, when in conflict, sharing your counterparts’ emotion may not arise spontaneously, but critically depend on also taking their perspective, to eventually promote helping. The fourth INTERACT objective is therefore to explain prosocial behavior by investigating the interplay of empathy- and ToM-related neural activity in interactive settings.

Taken together, these four objectives move beyond modular and passive accounts of social affect and cognition toward a dynamic and interaction focused perspective on empathy and ToM. In the following, we outline the multi method design of the INTERACT study, detailing how we operationalize these objectives across experimental tasks, neuroimaging modalities, and real life assessments.

## Methods and design

INTERACT will start by establishing a framework to systematically study the possible interrelation patterns in a relatively controlled manner by adapting a validated social affect and cognition task, the EmpaToM [16]. This adaptation will introduce dependencies between empathy and ToM, which enables probing their interplay and ***c****onjoint* effects (cEmpaToM, objective one). This lays the ground for increasing social interactivity in the following parts of the study. Enabling full social interactions necessarily reduces experimental control, but can approach naturalness, despite the constraints of neuroimaging, which we aim to achieve with an ***i****nteractive* EmpaToM version (iEmpaToM), and with complete interaction freedom in participants’ everyday lives using multi-agent EMA (objective two). While employment of both lab-based experimental tasks aims to test the range of possible patterns of interplay between empathy and ToM, EMA allows to study their natural occurrence in daily life. A within-person study design will allow linking everyday social interaction to brain function. To elucidate the role that free interplay of social affect and cognition plays in prosocial behavior (objective four), each of the previously mentioned tasks and measurement forms will include adapted measures of prosociality that will be jointly investigated. In the following, we detail the study design, ethical considerations of the study, participants and recruitment procedure, administered tasks, the multi-agent EMA protocol, questionnaire battery, data modalities that are assessed, and planned data analyses.

### Study design

**Fig. 2:**
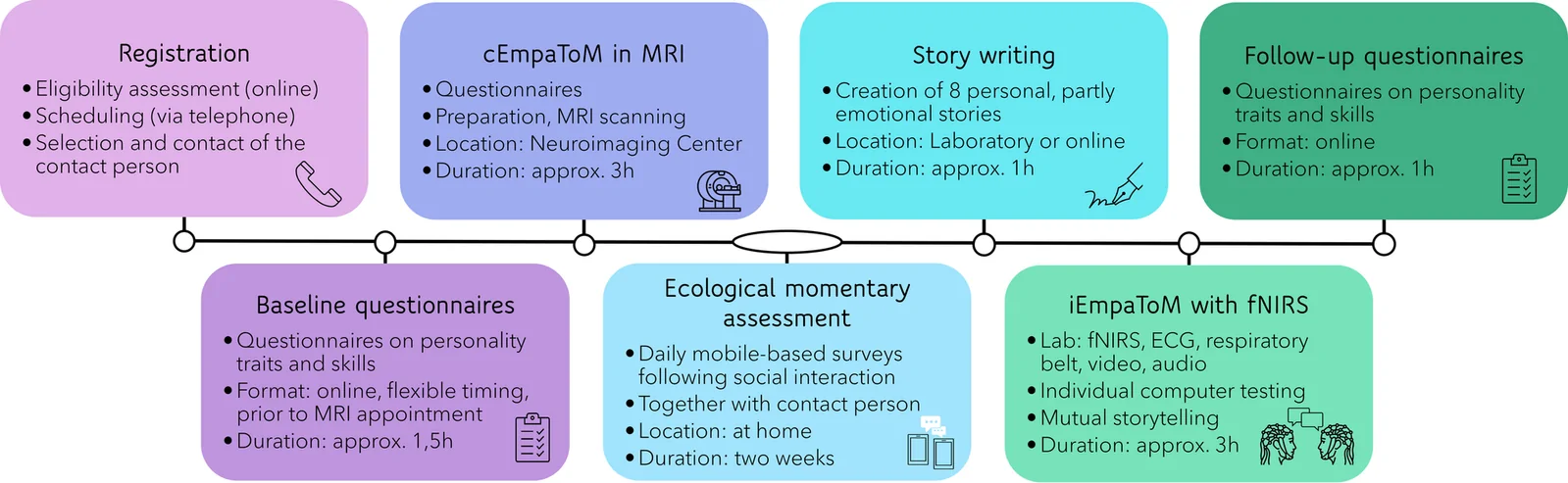
Timeline of the INTERACT project. After potential participants fill out an initial pre-screening questionnaire, an in-depth telephone call confirms eligibility criteria and identifies a potential partner participant. After confirming with the partner participant, target participants are selected for inclusion in the project (Registration). Before the initial lab session, target and partner participants fill out a set of questionnaires on relevant personality traits and skills (Baseline questionnaire). Then, target participants are invited to the first lab session, in which they perform the cEmpaToM task in the MRI, among others (cEmpaToM in MRI). After the first lab session, both target and partner participants perform a 14-day ecological momentary assessment (Ecological momentary assessment). After the 14-day ecological momentary assessment period, target participants are invited for the second lab session, in which they create the stimulus material for the upcoming final lab session together with study personnel (Story writing). In the final lab session, participants perform the iEmpaToM task together with another target participant unbeknownst to them (iEmpaToM with fNIRS). After the final lab session, both target and partner participants fill out a set of follow-up questionnaires (Follow-up questionnaires)

The multi-methodological nature of this study results in both a cross-sectional (cEmpaToM, iEmpaToM) and a longitudinal (multi-agent EMA) within-subjects study design. In short, participants will undergo three laboratory sessions, complete a 14-day EMA assessment together with a close contact person, and complete baseline and follow-up assessments of batteries of questionnaires investigating state- and trait-measures of related psychological functioning (e.g., trait measures of empathy, ToM, and prosociality). Figure 2 gives a graphical overview of the study workflow.

After initial registration for study participation and eligibility assessment via questionnaires and a telephone-based screening, participants (in the following: target participants) are invited to take part in the study together with a close contact person (such as their partner, family member or close friend; in the following: partner participants). Both target and partner participants are then asked to complete baseline questionnaires on personality traits and psychological processes relevant to the project’s overall objective (cf. Table 1). Target participants are subsequently invited to the first laboratory session, in which they provide informed consent for study participation, perform the cEmpaToM task in the MRI scanner (see “Tasks and Assessment Tools”), and are trained on the ensuing EMA sampling period. After the first laboratory session, both target and partner participants undergo a 14-day EMA sampling period. During the multi-agent EMA sampling period, target participants are invited for the second laboratory session. Here, the target participants prepare autobiographical stories as stimulus material for the upcoming third laboratory session together with the study personnel. The third laboratory session for target participants takes place after the end of the EMA sampling period (at least 20 days after the first laboratory session), wherein two target participants perform the iEmpaToM task during hyperscanning functional near-infrared spectroscopy (fNIRS) measurement. Finally, both target and partner participants complete a set of follow-up questionnaires similar to the baseline questionnaires.

**Table 1:** Overview of questionnaires applied at different time-points

Questionnaire name	Assessment time point				
	Baseline	MRI	EMA	iEmpaToM	Follow-up
Demographics	X				
Positive and Negative Affect Schedule (PANAS) [30]	X	X	X	X	X
Interpersonal Reactivity Index (IRI) [31]	X				X
Basic Empathy Scale (BES) [32]	X				X
Quality of Relationship Inventory (QRI) [33]	X				X
UCLA loneliness scale [34]	X				X
Social support questionnaire [German: Fragebogen zur Sozialen Unterstützung] (FSozU) [35]	X				X
Inventory for assessing interpersonal problems [Inventar zur Erfassung Interpersonaler Probleme] (IIP-32) [36]	X				X
Inclusion of Other in the Self (IOS) Scale [37]	X			X	
Paul van Lange Prosociality scale [38]	X				
Revised Adult Attachment Scale [39]	X				
Brief Self Control Scale (SCS-K-D) [40]	X				
Personality inventory for DSM-5 und ICD-11 (PID5CF + M) [41]	X				
Pittsburgh Sleep Quality Index (PSQI) [42]	X				
Brief symptom checklist (BSCL) [43]	X				
Social network index (SNI) [44, 45]	X				
Lifetime of experiences questionnaire (LEQ) [46, 47]	X				
Big Five Personality questionnaire (NEO-FFI-30 Short Form) [48]	X				
Multidimension assessment of interoceptive awareness version II (MAIA) [49]	X				
Prosociality scale [50]	X				
Autism-Spectrum Quotient - short version (AQ-K) [51]	X				
Social (interaction) anxiety: Social interaction anxiety scale (SIAS) [52, 53]	X				
Compassionate Engagement and Action Scale (CEAS-D) [54, 55]	X				
Quality of life (SF-36) [56, 57]	X				

Note. All questionnaires are administered in German language using German questionnaires, validated, or in-house translations

### Ethical consideration

This study has been approved by the ethics committee of TUD Dresden University of Technology (SR-EK-288062022). All participants grant informed consent prior to participation. They have the right to withdraw from the study at any point in time. The study is conducted in compliance with the Declaration of Helsinki.

### Participants and recruitment

Based on a power calculation informed by prior work [16, 29], INTERACT is recruiting *N* = 150 healthy individuals aged between 18 and 60 years. Exclusion criteria are: insufficient German language skills for task completion or understanding informed consent, current diagnosis of a mental disorder, ongoing psychotherapy or psychiatric medication, and no eligibility for MRI assessment (e.g., metal implants, severe cardiovascular disease). Moreover, targets must have a suitable close contact person willing to undergo the two-week EMA sampling period with them. Suitable partners have to be interacted with at least three times per week and have no mental disorder diagnosis. Participants are recruited using flyers, posters and online advertisements (e.g., via university-wide email list servers of TUD Dresden University of Technology).

### Tasks and assessment tools

#### cEmpaToM

We adapted the previously developed EmpaToM task [16] to assess the interplay of empathy and ToM ***c****onjointly* (termed cEmpaToM). In short, participants view video clips (~ 15s) in which narrators talk about purportedly autobiographical episodes; a bit like snippets cut from a conversation (for example scripts, see Table 2). These narrations are told in such a way that they induce both empathy (emotionally negative content of the stories) and prompt mentalizing (they are told in such a way that they necessitate the deduction of the mental state of the narrator). In contrast to the original EmpaToM task, the cEmpaToM experimentally manipulates the interaction pattern between empathy and ToM: the stories are designed so that empathy either facilitates or inhibits ToM, and vice versa (see Table 2). For example, a story might be told in such a way that correct mental state inference is required to empathize with a narrator, as correctly inferring another’s mental state changes the emotionality of a recounted story from emotionally neutral to negative, thus prompting empathy: “We had always so wished for that, and then she stands in front of me, telling me she’s pregnant … and all I can think of is that I just lost my job.” Correctly inferring the narrator’s mental state (that he is worried he might not be able to support a family) changes the content of the story, thus changing the emotionality of the story from neutral or even positive (pregnancy of his partner) to negative (worry about the financial situation). The stimulus set consists of 5 task conditions, with 12 videos per task condition: ToM facilitates empathy, ToM inhibits empathy, empathy facilitates ToM, empathy inhibits ToM, and a non-emotional control condition with no ToM demand.

**Table 2:** Patterns of dependency between empathy and ToM in the cEmpaToM task stories

Dependency	ToM facilitates emotion sharing	ToM inhibits emotion sharing	Emotion sharing facilitates ToM	Emotion sharing inhibits ToM
Example	We had always so wished for that, and then she stands in front of me, telling me she’s pregnant … *and all I can think of is that I just lost my job.*	I was so afraid to tell him I wanted to break up, he always gets so incredibly angry… *and then I come home and only find a note from him: “I’m sick of you”.*	Since my sister had the bike accident, she can’t walk without pain and it makes me so sad… *sometimes she already says I shouldn’t crowd her like that.*	My friend was beaten up in the metro just because of his dark skin. It makes me so angry and helpless… *but I also feel sorry for these guys with their broken lives.*
Explanation	After the context is given, *an abstract ToM cue makes the situation emotional.*	After the context is given, *an abstract ToM cue makes the situation less emotional.*	An emotional context makes *the following ToM demand* understandable.	An emotional context overrides the *following ToM demand.*

Note. This table describes hypothesized patterns of dependency between empathy and ToM in the cEmpaToM task. Example stories are given for each experimental condition, and explanations of how the stories represent the dependency patterns are provided

**Fig. 3:**
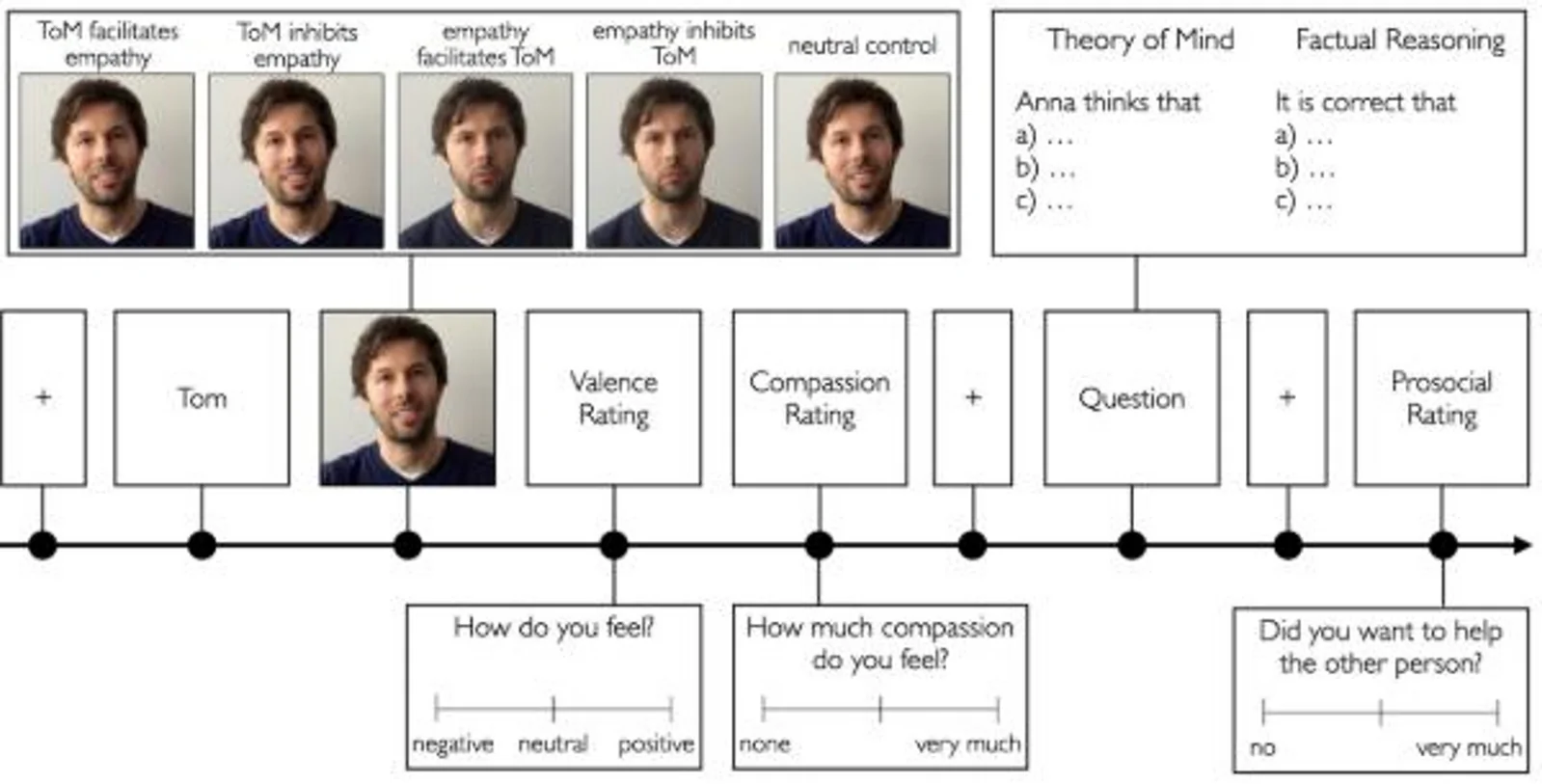
Example trial of the cEmpaToM task. Participants are presented with a short video clip (~ 15 s) of a narrator recounting an alleged autobiographical story. The stories in the experimental conditions are told in such a way, that Theory of Mind either facilitates or inhibits empathy, or empathy facilitates or inhibits ToM, while the stories in the neutral control condition are emotionally neutral and contain no ToM demand. Participants are then asked how they feel (valence rating, 4 s), and how much compassion they feel with the person (compassion rating, 4 s). They are then presented with a forced-choice question about the mental state of the narrator (ToM question, maximum of 15 s), or about the content of the story (factual reasoning question). Finally, participants are asked to rate how willing they are to help the narrator (prosocial rating, 4 s). All-in-all, the task contains 60 trials, with each condition being presented 12 times (12 different gender-matched narrators each present one story per condition), which is presented in two blocks of approximately 25 min each. All individuals whose images appear in the figure gave full consent for publication

After viewing the video, participants indicate via a rating scale how they feel (as a measure of affective sharing, 4 s) and how much compassion they feel (4 s). Then, ToM is probed via a question about the mental state of the person or, as a control condition, a factual reasoning question is asked (maximum response time: 15 s). At the end, participants rate how willing they are to help the person in the video (measure of prosociality, 4 s). This results in a 2 (interaction pattern: inhibitory, excitatory) x 2 (process driving interaction: ToM, empathy) experimental design, complemented by a neutral baseline condition (neutral emotionality, no ToM demand) to account, for instance, for narrator effects. To ensure conscientious task performance and mitigate potential fatigue due to the overall length of the task, the task is performed in two runs of 30 trials each (approximately 25 min per run). Videos are presented in a pseudo-randomized order, balancing the order of experimental conditions, interaction dependency (facilitatory, inhibitory), and potential narrator effects. The trial sequence is displayed in Fig. 3.

#### iEmpaToM

To test empathy and ToM in an actual social encounter we developed an ***i****nteractive* version of the EmpaToM (termed iEmpaToM) that embeds participants in a live interactive setting. Crucially, this interactive procedure allows participants to engage with each other in real time. For instance, they are able to ask for more information related to the story shared and to express care for the other. This setting transforms the partners from passive observers to (inter-)active social agents who can influence each other and become mutually coupled.

Each experimental session includes a dyad of two target participants who serve as experimental partners for each other. Prior to the experimental session, both target participants independently prepare to talk about eight situations from their past. These autobiographical narrations are prepared under the supervision of an experimenter. The narrations are either emotionally neutral or negative (i.e., sad) and either include ToM demands or not (Table 3). Two narrations are prepared per category.

In the iEmpaToM, the target participants’ task is to alternately talk about one of their prepared autobiographical situations. Each trial includes the following elements. First, one of the target participants tells one of the stories within a maximum of 35​ s. Then, both targets have 8​0 s to engage in free turn taking. At the end of each trial, participants provide self- and other-related ratings evaluating (a) their own and (b) their partner’s emotional state during the story, as well as during the free conversational phase (c, d) as a measure of affective sharing, (e) their own and (f) the partner’s compassion, (g) their own and (h) the partner’s degree of perspective-taking, (i) their urge to support the partner, and (j) the perceived support received from the partner as a measure of prosociality. The participants’ brain activity (fNIRS), respiration (breathing belt) and heart rate (electrocardiogram, ECG) are recorded continuously.

**Fig. 4:**
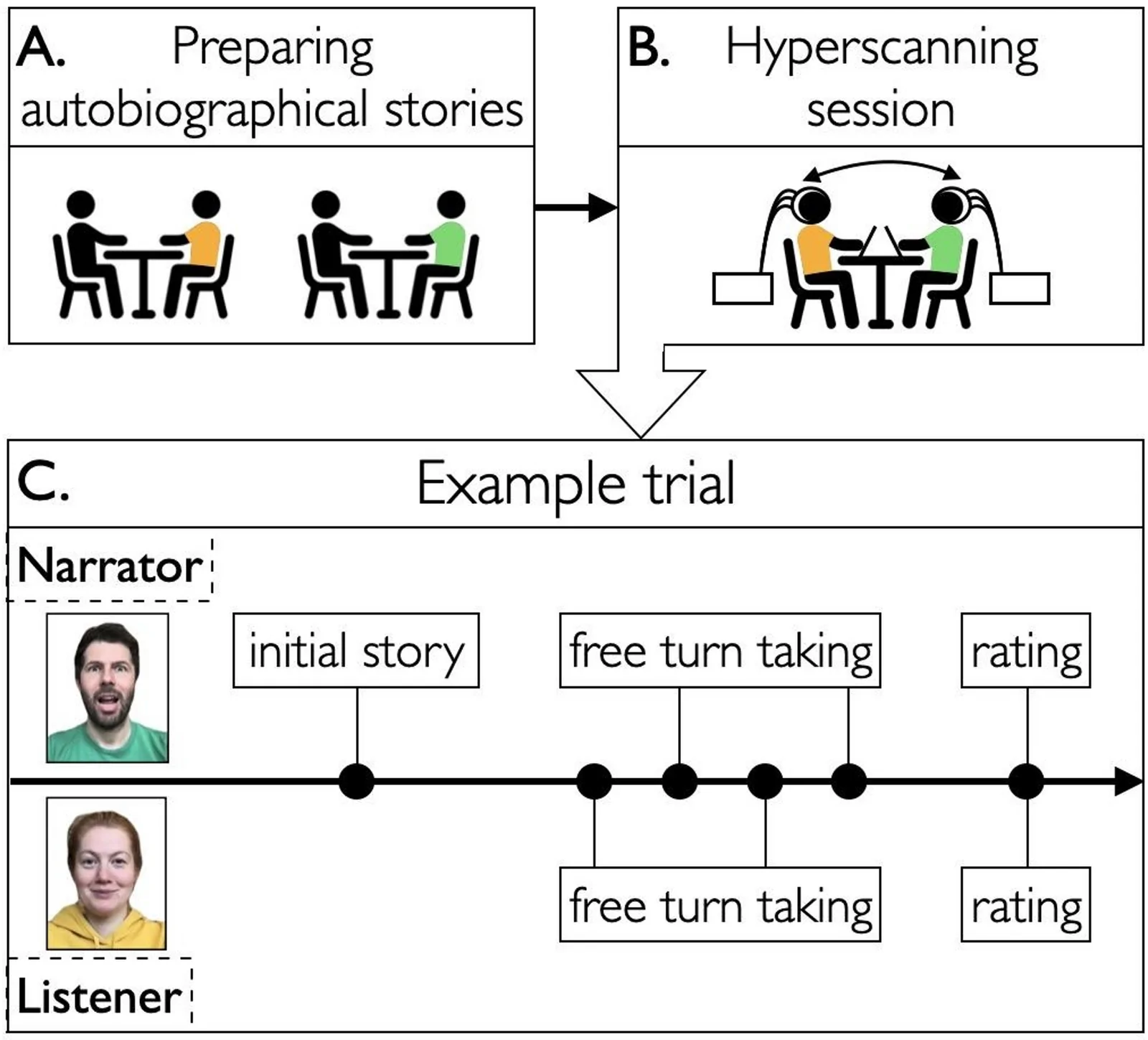
Procedure for the iEmpaToM session. (**A**) Prior to the experimental session, target participants independently prepare autobiographical stories under supervision of an experimenter. (**B**) Participants then engage in a mutual conversation in a hyperscanning setup while their brain activity is being recorded using fNIRS. (**C**) A trial starts with an initial autobiographical story by one participant, followed by a free turn taking and a rating of the emotional state (self/other) during the story telling/listening and the free turn taking, amount of compassion (self/other), engagement in perspective taking (self/other), felt support (by the other) and urge to support the other. All individuals whose images appear in the figure gave full consent for publication

Regarding the design, this setup results in a 2 (emotion: neutral, emotional) x 2 (ToM: without ToM demands, with ToM demands) x 2 (participant role: narrator, listener) design. The experimental sequence is displayed in Fig. 4.

**Table 3:** Examples of stories that target participants might tell during the iEmpaToM paradigm

Conditions	no ToM demand	ToM demand
Emotionally neutral	I go jogging several times a week, usually in the forest. I eat something beforehand for energy and always warm up first. I roll out my feet with a Spikeball, then do some stretches and activation exercises. I usually run distances ranging from 5 to 15 and sometimes I do longer runs on the weekend.	I used to watch my mom and grandma knit and always thought it was really cool, but I figured it wasn’t for me and would be too difficult. Plus, as a woman in her mid-20s, there seemed to be cooler hobbies. So I never dared to try it myself. But these baby socks tell a different story.
Emotionally negative	When I was about 12, one of my rabbits died which made me really sad. I really wanted rabbits as pets, so we got two, but unfortunately one died just a few weeks later for unknown reasons. I was heartbroken, especially because the other rabbit was now alone. My brother and I cried a lot. We then buried it in our garden.	It is really hard for me that my high school bestie and I have drifted apart so much. I miss her very much. I tried to talk it out with her, and she was understanding, but somehow things only got worse. Maybe we just needed that one big fight.

Note. Together with each target participant, two autobiographical stories per category are prepared

Directly following the iEmpaToM experiment, target participants perform a shortened version of the original EmpaToM task separately from each other (“control iEmpaToM”) [16] to assess differential effects of second-person (interacting with another person) and third-person (observing another person) perspectives. In this task, target participants are presented with a total of 16 video clips of four characters (two male, two female), each telling four different stories. The stories are either emotionally neutral or negative and either include ToM demands or not, resulting in a 2 (emotion: neutral, emotional) x 2 (ToM: without ToM demands, with ToM demands) experimental design. After viewing the video, ToM is probed via a question about the mental state of the person (ToM conditions) or a factual reasoning question (non-ToM conditions, maximum response time: 15 s). Then, participants indicate via a rating scale how they feel (as a measure of affective sharing), how much compassion they feel, their extent of perspective-taking, and how willing they are to help the person in the video (measure of prosociality). Videos are presented in a pseudo-randomized order, balancing the order of experimental conditions and potential actor effects.

#### multi-agent EMA

**Fig. 5:**
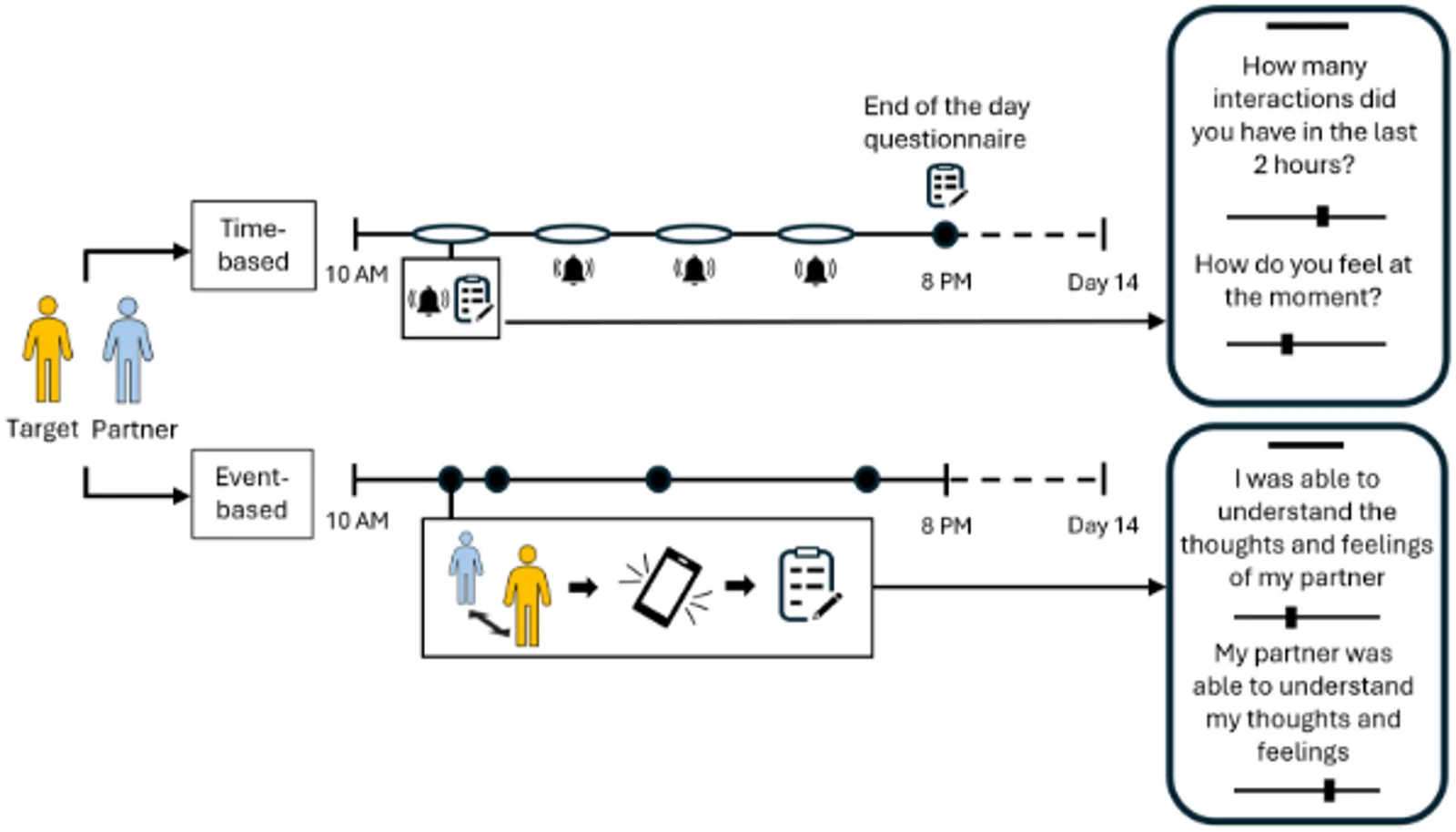
Overview of the two EMA sampling protocols. Time-based prompts are given at a random time within fixed time windows throughout the day. Event-based prompts are given after both target participants and partner participants were in close proximity for an extended amount of time. Both the target participant and the partner participant complete each questionnaire individually

Multi-agent EMA is implemented to extend laboratory-based investigations into real-world contexts in which social affect and cognition naturally co-occur. To obtain more objective measures of social affect and cognition in everyday life, target participants are recruited alongside one of their closest contact persons (e.g. romantic partners, family members, roommates, close friends). Target and partner participants use an app provided by us on their private smartphones to report on their social interaction in everyday life during a sampling period of 14 days (see Fig. 5). We apply two different assessment strategies in parallel: time-based and event-based sampling. For the time-based measurement, target and partner participants are prompted four times per day at random times within predefined one-hour windows between 10 a.m. and 8 p.m. (e.g., 10–11 a.m.) with one additional prompt at the end of each sampling day. Both participants are independently asked about their current mood and their most significant interaction that took place within the last two hours. They report on their own and the other’s presumed affect, thoughts, and prosocial behaviors. Additionally at the end of each sampling day the overall quality of social interactions that took place during the given day and overall affective state are assessed. For the event-based assessment, we use bluetooth-based proximity detection. If in close physical proximity for an extended period of time, both target and partner participants are probed with questions regarding their interaction. They are both asked about their own and the other’s presumed affect, thoughts, and prosocial behaviors during their previous interaction.

#### Questionnaires

For recruitment, interested target participants fill out an initial screening to evaluate eligibility including MRI compatibility, mental health, and the availability of a close contact person that would participate as the partner participant in the EMA assessment. Upon inclusion, target and partner participants fill out a questionnaire battery assessing personality traits, life events, socio-cognitive skills, social network characteristics, and medical details. At the beginning of the MRI and iEmpaToM session, target participant’s’ acute emotional states are assessed using the Positive and Negative Affect Schedule [30]. After completing all experimental sessions, a follow-up questionnaire battery similar to the baseline assessment is filled out again by both target and partner participants. Table 1 provides an overview of all administered questionnaires for target participants. Partner participants receive the same battery of baseline and follow-up questionnaires.

### Data modalities

#### *MRI assessment*

Brain activity during the cEmpaToM task is assessed using functional MRI. MRI data is acquired at the Neuroimaging Center at TUD Dresden University of Technology using a 3T Siemens MAGNETOM Prisma fit scanner (Siemens Healthcare, Erlangen, Germany) equipped with a 32-channel head coil.

Functional images are acquired using a multiband gradient-echo EPI sequence (TR = 780 ms, TE = 29 ms, flip angle = 56°, voxel size = 3 × 3 × 3 mm³, matrix size = 70 × 70, 48 slices, multiband factor = 4, phase encoding A→P). After a dual-echo gradient-echo B0 field map (TR = 544 ms, TE1/TE2 = 5.32/7.78 ms, flip angle = 49°, 50 slices, 3 × 3 × 3 mm³) the BOLD sequence is used for a noise-calibration scan (31 volumes), a resting-state run (454 volumes, 6 min), and a task-based run which is divided into two parts of approximately 25 min, with a break of at least 2 min in between.

Diffusion-weighted images are collected using a multiband spin-echo EPI sequence (TR = 3300 ms, TE = 66 ms, voxel size = 2 × 2 × 2 mm³, matrix size = 106 × 106, 72 slices, multiband factor = 3, acceleration factor = 2, 142 diffusion directions, multi-shell: b = 0, 1000, 2000, 3000 s/mm², 8 min), with an additional reverse phase-encoding b0 image for distortion correction. High-resolution T1-weighted structural images are collected using a 3D MPRAGE sequence (TR = 2400 ms, TE = 2.22 ms, TI = 1030 ms, flip angle = 8°, voxel size = 0.8 × 0.8 × 0.8 mm³, matrix size = 320 × 300 × 208, GRAPPA factor = 2, acquisition time = 6:38 min).

#### fNIRS assessment

**Fig. 6:**
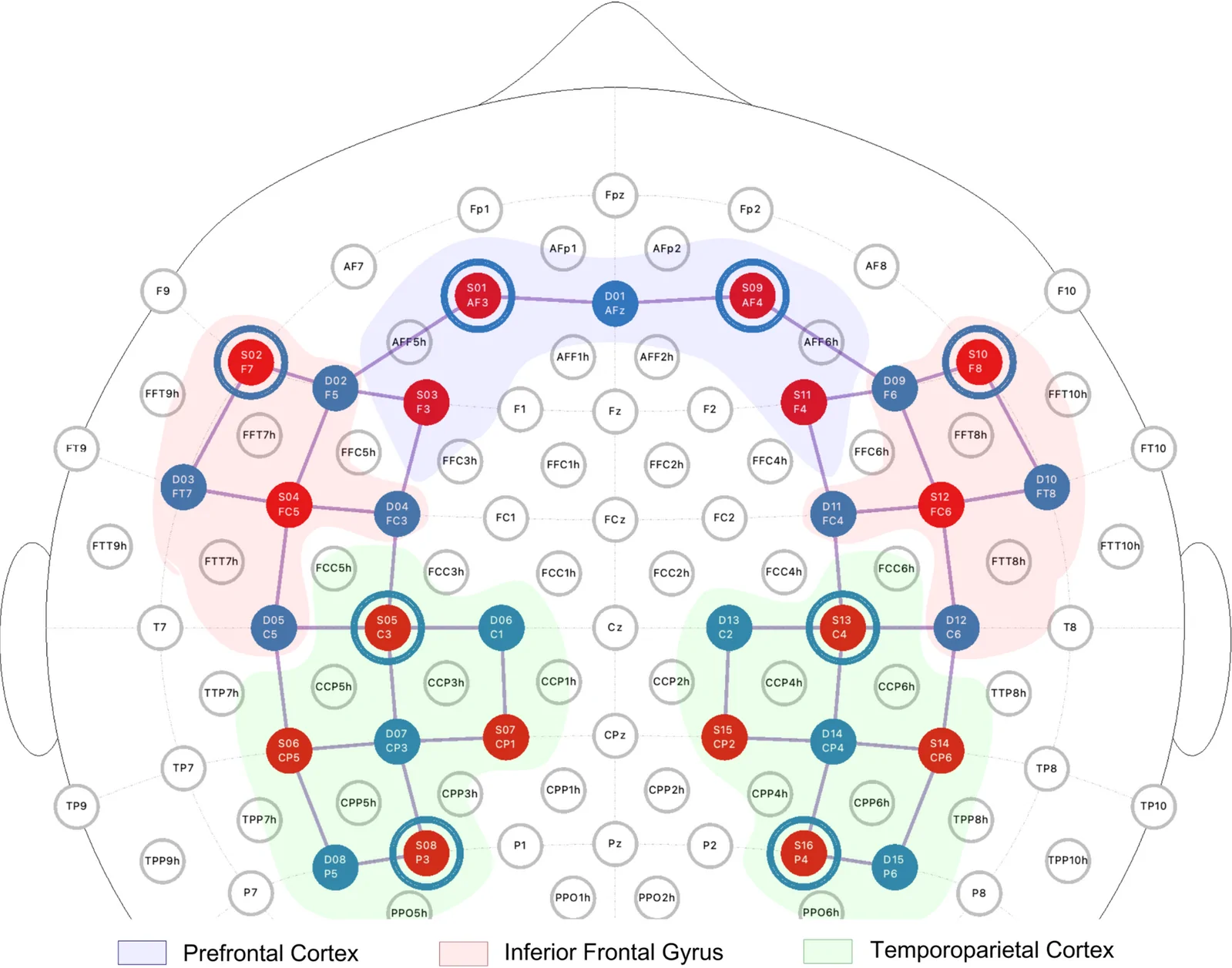
fNIRS optode configuration and ROI definition. Topographical representation of the fNIRS montage. Red and blue markers indicate sources and detectors, respectively. Connecting lines indicate measurement channels, and blue circles indicate short-separation channels. Shaded regions cover the predefined regions of interest (ROIs): prefrontal cortex (PFC), inferior frontal gyrus (IFG), and temporoparietal cortex (TPC)

Brain activity during iEmpaToM is measured using two NIRSport2 16 × 16 devices (NIRx Medizintechnik GmbH, Germany). Data is acquired via Hyperscan and Aurora software (NIRx Medizintechnik GmbH, Germany). The devices capture near-infrared light at 760 and 850 nm, corresponding to deoxyhemoglobin (HbR; reduced hemoglobin) and oxygenated hemoglobin (HbO). To optimally capture neural activity, the setup maintains a consistent source-detector distance of 25–35 mm and a sampling rate of 10.9 Hz. Optode locations were defined according to the 10–20 international electroencephalography (EEG) system, with corresponding brain regions identified using the fNIRS Optodes’ Location Decider (fOLD) toolbox. The customized montage comprising 16 sources, 15 detectors, and 8 short-separation detectors was designed in NIRSite, covering three regions of interest (ROIs): the inferior frontal gyrus, prefrontal cortex, and temporoparietal cortex (including the temporoparietal junction, Fig. 6). The montage yields 50 channels, with 8 short-distance channels for physiological noise correction. The same configuration is applied to both target participants in each dyad. The duration of assessment is around 1.5 h.

#### Peripheral physiological recordings

Peripheral physiological recordings are acquired across the neuroimaging paradigms (cEmpaToM, iEmpaToM, iEmpaToM control task) to index autonomic and respiratory dynamics. During the fMRI scan (i.e., cEmpaToM task), peripheral physiological signals are recorded using the BrainAmp ExG MR and respiration belt MR Brain Products GmbH. ECG is recorded using a bipolar chest configuration with MR-conditional electrodes positioned to maximize reliable R-wave detection while minimizing scanner-related artifacts. In line with MR acquisition procedures, electrodes are applied following skin preparation and impedance verification, and leads will be routed in a straight, loop-free configuration close to the bore midline to reduce both heating risk and motion-related contamination. Respiratory activity is monitored using an MR-compatible respiration belt positioned around the thorax or abdomen, depending on the participant’s predominant breathing pattern, to capture respiratory patterns during scanning. Physiological recordings are synchronized with the MRI acquisition to permit precise temporal alignment with image volumes and task events.

During the iEmpaToM paradigm, peripheral physiological signals are acquired using the WINGS2 device integrated with the NIRSport2 system (NIRx Medizintechnik GmbH, Germany). ECG is recorded using an ExG channel in a bipolar chest configuration to provide a continuous measure of cardiac activity, while respiration is recorded using a respiration sensor, implemented as an inductive respiration belt positioned around the thorax or abdomen to capture breathing patterns across task blocks. ECG, respiration, fNIRS, and task triggers are recorded in a synchronized acquisition stream within the NIRx environment, allowing precise temporal alignment with interaction blocks, narrator/listener role transitions, and post-interaction rating periods.

#### Video and audio recordings

The interactive task sessions (iEmpaToM) are recorded using the Mangold VideoSyncPro multimedia recording system [58]. The setup consists of 3 PTZ Dome FullHD cameras filming participants’ faces frontally and from the side, 2 reverse radio microphones and one boundary layer microphone all connected to a multimedia workstation via an audio interface and a 19-inch rack system. All components are connected through a dedicated wireless network infrastructure to ensure stable and synchronized recording of audio and video streams during the experiment.

#### multi-agent EMA

Repeated self-report questionnaire data are collected via participants’ private smartphones using the m-path platform (https://m-path.io; [59]) on both Android (4.8.1) and iOS (1.1.1) systems. As described above, for the event-based sampling we collect proximity data between targets and interaction partners. In order to obtain a strong Bluetooth signal the partners of each dyad are asked to carry a Bluetooth Sensor (BLE Location Beacon) throughout the day. The sensor is connected to the smartphone of the target participant. This enables a stable and precise measurement of proximity data via Bluetooth Signal Strength. We also collect GPS coordinates as objective indicators of co-presence and interaction duration.

### Data analysis approach

Analyses will follow a unified, multimodal framework that links behavioral, neural, physiological, and EMA data to test our hypotheses about interplay between empathy and ToM and their relation to prosocial behavior. Analyses will be conducted at group and individual levels, and will combine confirmatory hypothesis testing with exploratory, data‑driven approaches. Primary inferential strategies include general linear models for task and imaging data, mixed‑effects (multilevel) models for repeated and nested measures (including dyadic Actor-Partner Interdependence Models for EMA and interaction data), and time‑series / connectivity methods to assess directed and undirected coupling within and between brains and bodily signals. For fMRI data we will use standard preprocessing and GLM-based contrasts to evaluate condition effects, complemented by effective connectivity analyses to probe directional influences between networks. Diffusion MRI data will be analyzed using tractography approaches to assess structural connectivity across the whole brain (tractogram) as well as between regions of interest. For fNIRS hyperscanning we will analyze within‑brain activation with mixed‑effects models and quantify inter‑brain synchrony using coherence/wavelet or related metrics, with permutation tests against surrogate dyads to establish specificity. Physiological signals (ECG, respiration) will be preprocessed to derive heart rate, heart rate variability, respiratory metrics and used both as covariates and as variables in synchrony and brain–body coupling analyses. Video and audio data will be analyzed using automated transcription and facial recognition methods to quantify semantic and syntactic properties of spoken language as well as facial expression in relation to direct social interaction. Behavioral and questionnaire outcomes will be modeled with repeated‑measures and multilevel regression to assess within- and between-person effects and to link lab indices to EMA measures. Where appropriate, predictive and multivariate techniques (e.g., cross-validated regression or classification) will be applied to test whether neural and physiological markers predict prosocial decisions or real‑world interaction patterns.

Throughout, we will control for confounds (e.g., motion, baseline affect, demographic variables), correct for multiple comparisons using appropriate familywise or false discovery rate procedures, and report effect sizes and confidence intervals. Exact preprocessing steps, statistical models, model specifications, covariates, region-/channel-wise definitions, thresholding, and correction procedures will be detailed in study‑specific analysis plans.

### Preregistration and transparency

Each publication arising from this project will be preregistered prior to data analysis, specifying the hypotheses, outcome measures, detailed analysis pipelines, and planned sensitivity checks. All analysis code, anonymized data and study materials will be made available in open repositories to maximize transparency and reproducibility.

## Discussion

Despite globalizing societies, loneliness and isolation constitute the biggest mental and public health challenges [60]. At the same time, the quality and number of social interactions are among the best predictors of long-term health [61]. Integrating these findings, it seems imperative to gain a better understanding of how successful and engaging social interactions come about. However, our understanding of the mechanisms that enable adaptive social behavior is limited by simplified concepts and impoverished methodological approaches: Processes that enable social interaction are often viewed as isolated building blocks; participants are treated as passive observers of social stimuli [20].

INTERACT takes on the challenge to investigate interacting processes of affect and cognition in real interaction between people. To this end, we apply novel and promising methods reaching from neuroscience to economics and ambulatory research. Particularly, we study (1) patterns of interplay between empathy- and ToM-related neural networks in (2) both, observational contexts and actual, full social interactions using fMRI and fNIRS; and relating it to participants’ actual social lives assessed with multi-agent EMA. This lays the groundwork for (3) explaining prosocial behavior and, in the future, (4) improve social functioning by training empathy and ToM.

The aims of the INTERACT study are ambitious and progressive. However, the conceptual breadth and ambition of the project also give rise to several limitations that should be considered carefully. A central aspect here is the inherent trade-off between ecological validity and experimental control [62, 63]. To ensure sufficient control over conversational content during social interactions, we designed a structured interaction paradigm that systematically manipulates affective (i.e., empathy) and cognitive (i.e., ToM) processes, which may reduce spontaneity and authenticity [64]. Furthermore, although we aim for actual, full social interaction, experimental operationalizations cannot capture the full *Gestalt* of social interaction but will necessarily capture only a subset of variables. These limitations are not unique to INTERACT but reflect broader challenges in social interaction research [62, 65]. Nevertheless, we believe that the proposed design achieves a good balance between ecological validity and experimental control, allowing for meaningful insights while preserving interpretability across the more experimentally constrained elements of the overall study. Successful completion of the INTERACT project will provide ample opportunities for the development of research beyond the projects’ scope. For example, we take an important step away from a modularized approach toward a complex-systems approach to studying brain function. This integration of functions could also be applied to more basic perceptual (e.g., biological motion and person perception) or motivational processes (e.g., affiliation or compassionate care), further expanding this integrative framework of social brain functioning. We will assess and openly share a rich dataset encompassing comprehensive dimensions of social cognition, enabling the testing of diverse hypotheses beyond the aims of the present project.

If successful, INTERACT could contribute to a paradigm shift towards truly interactive neuroscience, generating insights to help address mental and public health challenges posed by loneliness and isolation and ultimately improve long-term health.

## Data Availability

No datasets were generated or analysed during the current study.

## Abbreviations

EMA: Ecological momentary assessment
DMN: Default mode network
DTI: Diffusor tensor imaging
CN: Control networks
MRI: Magnetic resonance imaging
fMRI: functional magnetic resonance imaging
fNIRS: functional near-infrared spectroscopy
ToM: Theory of Mind
ECG: electrocardiogram
